# Creating a multifaceted prognostic model for cutaneous melanoma: the convergence of single-cell and bulk sequencing with machine learning

**DOI:** 10.3389/fcell.2024.1401945

**Published:** 2024-05-06

**Authors:** Fei Mao, Neng Wan

**Affiliations:** ^1^ Department of Urology, The Affiliated Huaian No. 1 People’s Hospital of Nanjing Medical University, Huai’an, China; ^2^ Department of Plastic Surgery, The Affiliated Huaian No. 1 People’s Hospital of Nanjing Medical University, Huai’an, China

**Keywords:** cutaneous melanoma, single-cell sequencing, bulk-RNA, machine learning, inflammation

## Abstract

**Background:**

Cutaneous melanoma is a highly heterogeneous cancer, and understanding the role of inflammation-related genes in its progression is crucial.

**Methods:**

The cohorts used include the TCGA cohort from TCGA database, and GSE115978, GSE19234, GSE22153 cohort, and GSE65904 cohort from GEO database. Weighted Gene Coexpression Network Analysis (WGCNA) identified key inflammatory modules. Machine learning techniques were employed to construct prognostic models, which were validated across multiple cohorts, including the TCGA cohort, GSE19234, GSE22153, and GSE65904. Immune cell infiltration, tumor mutation load, and immunotherapy response were assessed. The hub gene STAT1 was validated through cellular experiments.

**Results:**

Single-cell analysis revealed heterogeneity in inflammation-related genes, with NK cells, T cells, and macrophages showing elevated inflammation-related scores. WGCNA identified a module highly associated with inflammation. Machine learning yielded a CoxBoost + GBM prognostic model. The model effectively stratified patients into high-risk and low-risk groups in multiple cohorts. A nomogram and Receiver Operating Characteristic (ROC) curves confirmed the model’s accuracy. Low-risk patients exhibited increased immune cell infiltration, higher Tumor Mutational Burden (TMB), and potentially better immunotherapy response. Cellular experiments validated the functional role of STAT1 in melanoma progression.

**Conclusion:**

Inflammation-related genes play a critical role in cutaneous melanoma progression. The developed prognostic model, nomogram, and validation experiments highlight the potential clinical relevance of these genes and provide a basis for further investigation into personalized treatment strategies for melanoma patients.

## Introduction

Cutaneous melanoma, a malignant neoplasm originating from melanocytes, represents a formidable challenge in the realm of cancer research and clinical management (Marzagalli et al., 2019; Long et al., 2023; Patel et al., 2023). With its aggressive nature and propensity for metastasis, melanoma is responsible for a disproportionate number of skin cancer-related deaths worldwide (Arnold et al., 2022). Despite advancements in early detection and therapeutic approaches, a significant proportion of melanoma patients still face poor prognoses, necessitating the development of novel and more precise prognostic tools (Barry et al., 2018; Granata et al., 2022; Perez et al., 2022).

The tumor microenvironment (TME) plays a pivotal role in the progression and outcome of melanoma. Within this complex ecosystem, various cellular and molecular components interact dynamically to shape the course of the disease (Lee and Cheah, 2019; Chen et al., 2021; Xu et al., 2022). Among these factors, inflammation has emerged as a central player, influencing tumor initiation, progression, and response to treatment (Jin and Jin, 2020). The presence of chronic inflammation in the TME is associated with increased tumor invasiveness, immune evasion, and resistance to therapy, all of which contribute to poorer patient outcomes (Iyengar et al., 2016). Thus, understanding the inflammatory landscape within melanoma TME holds great promise for improving prognostication and treatment strategies.

The advent of multiomics technologies has revolutionized our ability to decipher the intricate biology of melanoma and its microenvironment (Newell et al., 2022). Among these techniques, single-cell sequencing has garnered significant attention due to its capacity to unveil the heterogeneity and complexity of the TME at an unprecedented resolution (Huuhtanen et al., 2023). By profiling individual cells within the tumor, single-cell sequencing provides insights into the diverse cellular populations, their functional states, and the intricate molecular crosstalk that underlies melanoma progression (Naulaerts et al., 2023). Bulk sequencing, on the other hand, offers a comprehensive view of genetic alterations and gene expression profiles across the entire tumor, enabling a broader understanding of the genomic landscape (Wang et al., 2022). Integrating these multiomics approaches with advanced machine learning techniques presents an exciting opportunity to construct robust prognostic models that can capture the interplay between inflammation and melanoma progression.

However, while the potential of multiomics and machine learning approaches in melanoma research is promising, it is essential to acknowledge their limitations. Data integration from disparate sources can be challenging, necessitating careful preprocessing and harmonization to ensure the validity of findings. Additionally, the dynamic nature of the TME poses a significant challenge in capturing its complexity accurately. Moreover, despite their predictive power, prognostic models developed through bioinformatics analysis may require validation in independent cohorts to establish their clinical utility.

In this study, we present a novel approach that combines single-cell sequencing analysis, bulk sequencing analysis, and machine learning to construct an inflammation-related prognostic model in cutaneous melanoma. By leveraging the strengths of these technologies, we aim to gain a deeper understanding of the role of inflammation in melanoma progression and develop a robust prognostic tool that can guide clinical decision-making. Through this integrated approach, we hope to contribute to the growing body of knowledge in melanoma research and advance towards more effective strategies for the management of this challenging malignancy.

## Materials and methods

### Bulk RNA-seq data download

In this study, a total of four bulk sequencing datasets for cutaneous melanoma were downloaded and utilized. These datasets include The Cancer Genome Atlas (TCGA) cohort, GSE19234 cohort, GSE22153 cohort, and GSE65904 cohort (Bogunovic et al., 2009; Jönsson et al., 2010; Cabrita et al., 2020). The “sva” method was applied to standardize the downloaded bulk data, effectively mitigating any noticeable batch effects. Additionally, we matched gene expression data with clinical survival data. Log2 transformation was uniformly applied to all the data.

### Single cell-seq data download

The single-cell sequencing data for cutaneous melanoma (GSE115978) was obtained from the GEO database (https://www.ncbi.nlm.nih.gov/geo/) in count format (Jerby-Arnon et al., 2018). To standardize this data, both the “LogNormalize” and “vst” methods were applied. High variable genes were selected using the “FindVariableFeatures” function. Subsequently, PCA (Principal Component Analysis) was performed for dimension reduction, and the batch effects were mitigated using the harmony function. Cell types were annotated based on highly expressed markers within each cluster, and visualization was carried out using t-SNE and UMAP techniques.

### Source of inflammation-related genes

Inflammation-related genes were sourced from the Genecards database (https://www.genecards.org). Using the search bar, “inflammation” was entered as the keyword for retrieval. The top 100 genes ranked by their scores were exported (Supplementary Table S1). These genes are considered highly relevant to inflammation and will be used for subsequent analysis.

### Cells were scored on inflammation-related genes at the single-cell level

The gene expression score was initially computed for the melanoma single-cell sequencing data GSE115978 using the “AUCell” R package. Following this, the 100 genes related to inflammation were extracted to derive inflammation-related area under the curve (AUC) scores. These scores were then divided into two groups, namely, the high-AUC group and the low-AUC group, based on the median value. Differential gene expression analysis was subsequently conducted to identify genes that exhibited differential expression between these two groups. To explore the differential enrichment pathways between the two groups, Gene set variation analysis (GSVA) analysis was employed.

### Spearman correlation analysis on inflammatory genes in single cell sequencing data

Once the individual cell inflammatory AUC scores were acquired, the “cor.test” function was employed to perform Spearman correlation analysis to identify genes correlated with these AUC scores. Subsequently, the first 150 genes found to be associated with the AUC scores were intersected with the differentially expressed genes identified between the two AUC groups. This final outcome was regarded as the most pertinent set of genes related to inflammation in the single-cell sequencing data.

### Weighted gene coexpression network analysis (WGCNA)

In this study, we conducted a Weighted Gene Co-expression Network Analysis (WGCNA) of a set of 100 inflammation-related genes on TCGA melanoma dataset. Following data normalization, we constructed a co-expression network using the “WGCNA” R package, identified gene modules, and assessed their correlation with inflammation-related traits. Hub genes within modules were determined based on intramodular connectivity, and functional annotation was performed to elucidate the biological processes associated with these modules. This analysis aimed to uncover co-expression patterns, potential key regulators, and functional insights related to inflammation in the TCGA dataset. Finally, we intersected the key genes in the key modules obtained by WGCNA with the key genes in the single-cell sequencing data, and the remaining genes were used to construct a prognostic model.

### Machine learning to construct prognostic models

In this study, we employed a multi-step approach to construct prognostic models using a selected set of genes derived from unifactor COX regression analysis on the TCGA dataset and applied machine learning techniques in three independent GEO cohorts. First, unifactor COX regression analysis was conducted on the TCGA dataset to identify genes strongly associated with prognosis. Subsequently, a variety of machine learning algorithms, including RSF, COXBoost, Enet, GBM, Lasso, plsRcox, Ridge, StepCox, and Survivor-SVM, were utilized individually and in combinations to construct prognostic models (Wang Q. et al., 2023; Wang D. et al., 2023; Pei et al., 2023). During the construction of the model, the TCGA cohort is used as the training set and the three GEO cohort is used as the validation sets. The Concordance Index (C-index) comparison of the models is performed in validation sets. To assess the performance of these models, the C-index was calculated for each model, ranking them from high to low. The “ComplexHeatmap” package in R was used to visualize the results, providing a comprehensive overview of the prognostic model performance across different algorithms and their combinations. This analysis aimed to identify the most effective models for predicting prognosis in the context of the three GEO cohorts, with the C-index serving as a key metric for evaluation.

### Evaluation and validation of prognostic models

The prognostic model was applied to multiple cohorts, including the TCGA cohort, GSE19234, GSE22153, and GSE65904. For each patient in these cohorts, a risk score was computed. Subsequently, patients were categorized into high-risk and low-risk groups based on the median value derived from these four cohorts. To assess survival outcomes, we utilized the Kaplan-Meier method to generate survival curves and conducted log-rank tests to establish statistical significance. Furthermore, we developed a nomogram that integrates the risk scores with various clinical characteristics to predict survival probabilities at 1, 3, and 5 years. To gauge the nomogram’s accuracy, we constructed calibration curves. Additionally, we evaluated the model’s robustness by generating ROC curves and calculating the area under the curve (AUC).

### Analysis of immunity and mutation in tumor microenvironment

To gain deeper insights into the prognostic model’s relevance in the tumor microenvironment, we conducted immune and mutational analyses, along with predicting differences in immunotherapy response between the high-risk and low-risk groups. These analyses were carried out within the TCGA cohort. Initially, we divided patients in the TCGA cohort into high-risk and low-risk groups based on their risk scores, which were previously calculated. Next, we obtained data regarding immune cell infiltration and matched patient groups with their respective immune cell infiltration profiles. The results were then visualized using the “pheatmap” R package. Similarly, we calculated the tumor mutation load (TMB) in a manner analogous to the immune cell infiltration analysis. TMB data from the TCGA cohort were linked with the risk score groups to identify genes with high mutation rates. Visualization was accomplished using the “ComplexHeatmap” package. Furthermore, we utilized the “estimate” R package to compute immune scores, stromal scores, Estimate scores, and tumor purity for both the high-risk and low-risk groups. To evaluate the potential efficacy of immunotherapy, we employed immunotherapy data from the TCIA database to predict TCIA scores for the high-risk and low-risk groups, thereby assessing differences in immunotherapy response.

### Cell culture and transfection

The human malignant melanoma cell lines, namely, A375 and WM115, were sourced from the cell repository established by the Type Culture Collection of the Chinese Academy of Sciences. These cell lines were cultured in DMEM (Gibco BRL, Rockville, MD, United States), complemented with 10% fetal bovine serum (FBS, Gibco BRL, Rockville, MD, United States), and a 1% penicillin-streptomycin solution. Incubation was carried out at 37°C in a 5% CO_2_ environment.

To conduct gene knockdown experiments, small interfering RNA (siRNA) and its corresponding negative control (NC) were custom-synthesized on our behalf by Ribobio, based in Guangzhou, China. The specific siRNA sequences targeting STAT1 can be found in Supplementary Table S2. Subsequently, we performed transfections on A375 and WM115 cells using Lipofectamine 3000, following the manufacturer’s instructions provided by Invitrogen, situated in Carlsbad, CA, United States.

### CCK8, clony formation, transwell assays

The evaluation of cell proliferation in both A375 and WM115 cell lines involved the utilization of Cell Counting Kit 8 (CCK-8). Following transfection, cells were seeded into 96-well plates overnight, with each well containing 2000 cells (maintained at 37°C under 5% CO_2_). Subsequently, a mixture comprising 10 µL of CCK-8 labeled reagent (A311-01, Vazyme, Nanjing, China) and 90 µL of serum-free medium was added to the cells. They were then incubated in darkness at 37°C for 2 h, and the absorbance at 450 nm was measured using an enzyme marker (A33978, Thermo, Waltham, MA, United States).

For the clony formation assays, transfected A375 and WM115 cells were cultured in 6-well plates for approximately 12 days. Following this incubation period, cells were subjected to staining with 0.1% crystal violet for 30 min, followed by a PBS wash. The colonies exceeding 1 mm in diameter were then enumerated.

Invasion and migration assays were executed by employing a 24-well transwell chamber (BD Biosciences, Franklin Lakes, NJ, United States). In the upper chamber, A375 cells were suspended in 200 µL of serum-free medium, while the lower chamber contained 600 µL of medium supplemented with 10% fetal bovine serum. After incubating for 48 h, the cells that had penetrated the upper membrane surface were meticulously removed. The remaining cells were fixed with 4% paraformaldehyde and subjected to staining with 0.1% crystal violet (Solarbio, Beijing, China). Following this, three distinct fields of view were observed and quantified under a light microscope at ×200 magnification.

### Statistical analysis

In R software (version 4.3.2), the analysis involved several steps. Single-cell analysis was executed using the Seurat package, while the assessment of gene heterogeneity associated with inflammation was conducted through the AUCell method. Highly correlated genes were identified using Spearman correlation analysis, and modules linked to inflammation were pinpointed using the WGCNA method. Prognostic models were constructed using machine learning techniques such as RSF, COXBoost, Enet, GBM, Lasso, plsRcox, Ridge, StepCox, and survival-SVM. Survival analysis was performed using the Kaplan-Meier (K-M) method, and the robustness of the models was assessed using ROC curves. Throughout the study, all experiments were rigorously repeated three times to ensure the reliability of the findings, and statistical significance was determined with a threshold of *p* < 0.05.

## Results

### Single-cell sequencing analysis identified the heterogeneity of inflammation-related genes in cutaneous melanoma

**FIGURE 1:**
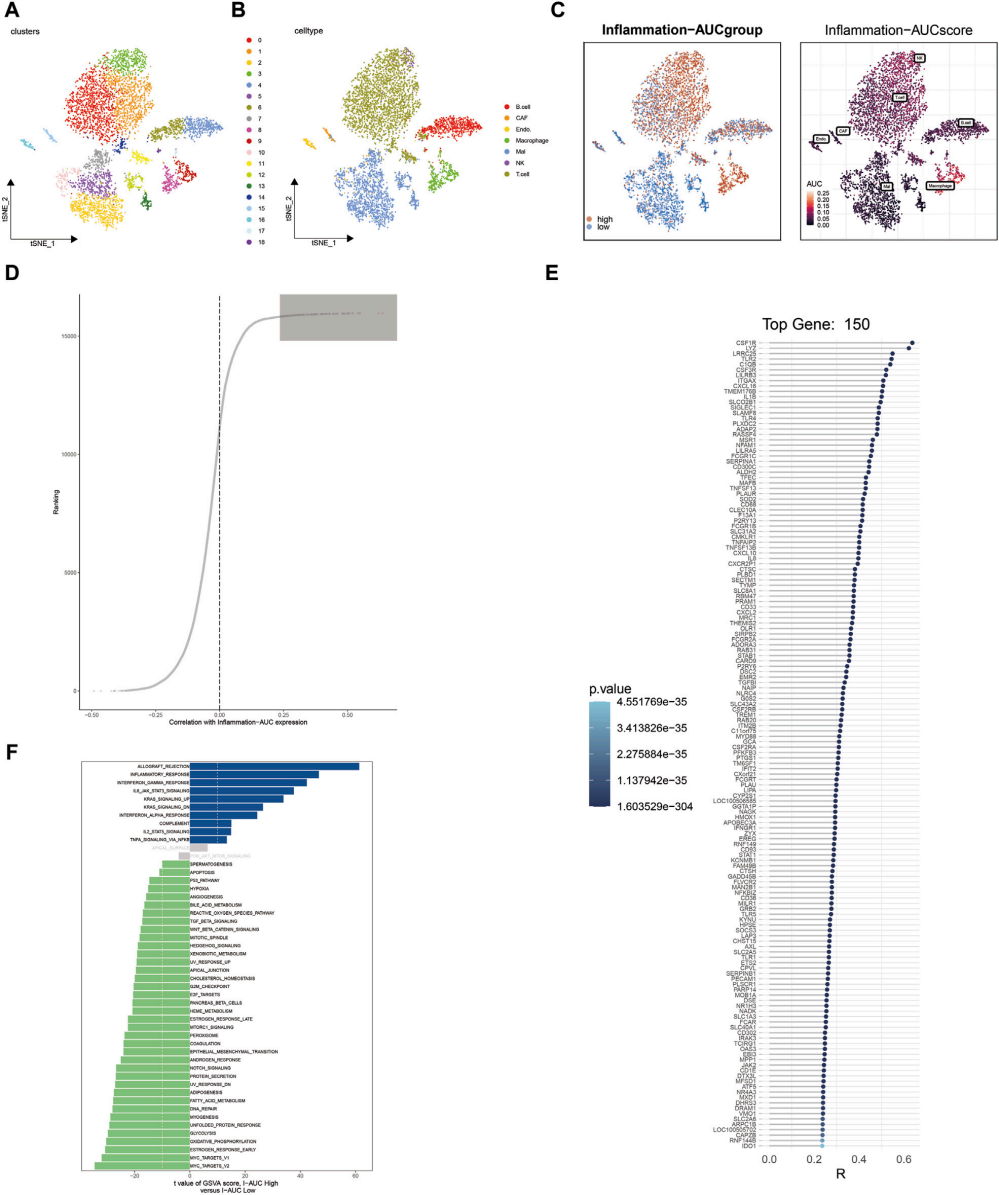
Single-cell sequencing analysis identifies heterogeneity in inflammation-related genes in cutaneous melanoma. **(A)** t-SNE dimensionality reduction clustering of single-cell data into 19 clusters. **(B)** Annotation of cell clusters, revealing cell types including B cells, CAFs, endothelial cells, macrophages, Mal cells, NK cells, and T cells. **(C)** Identification of differentially expressed genes between the high-AUC and low-AUC groups. **(D,E)** Spearman correlation analysis identifies the top 150 genes correlated with inflammatory AUC scores. **(F)** GSVA analysis reveals differential enrichment pathways between high-AUC and low-AUC groups.

Following harmony batch removal and tSNE dimensionality reduction clustering, the entire cell population was segregated into 19 clusters, as depicted in Figure 1A. Subsequently, through the annotation of characteristic genes within these clusters, they were identified as B cells, cancer-associated fibroblasts (CAFs), endothelial cells, macrophages, malignant (Mal) cells, NK cells, and T cells, as illustrated in Figure 1B. To assess the activation of inflammation-related genes at the single-cell level, AUCell analysis was employed, classifying cells into either the high-AUC group or the low-AUC group based on the median value. Notably, NK cells, T cells, and macrophages exhibited elevated inflammation-related scores. Further analysis involved the extraction of differentially expressed genes between the high-AUC and low-AUC groups, as depicted in Figure 1C. Spearman correlation analysis was conducted to pinpoint the top 150 genes strongly associated with the inflammatory AUC score, as showcased in Figures 1D, E. Subsequently, 67 genes were selected for further analysis through the intersection with the pool of differentially expressed genes. The identification of differentially activated pathways between the high-AUC and low-AUC groups was accomplished using GSVA analysis, encompassing pathways such as allograft rejection and inflammatory response, as presented in Figure 1F.

### Weighted Gene Coexpression Network Analysis (WGCNA) identifies key inflammatory modules in cutaneous melanoma

**FIGURE 2:**
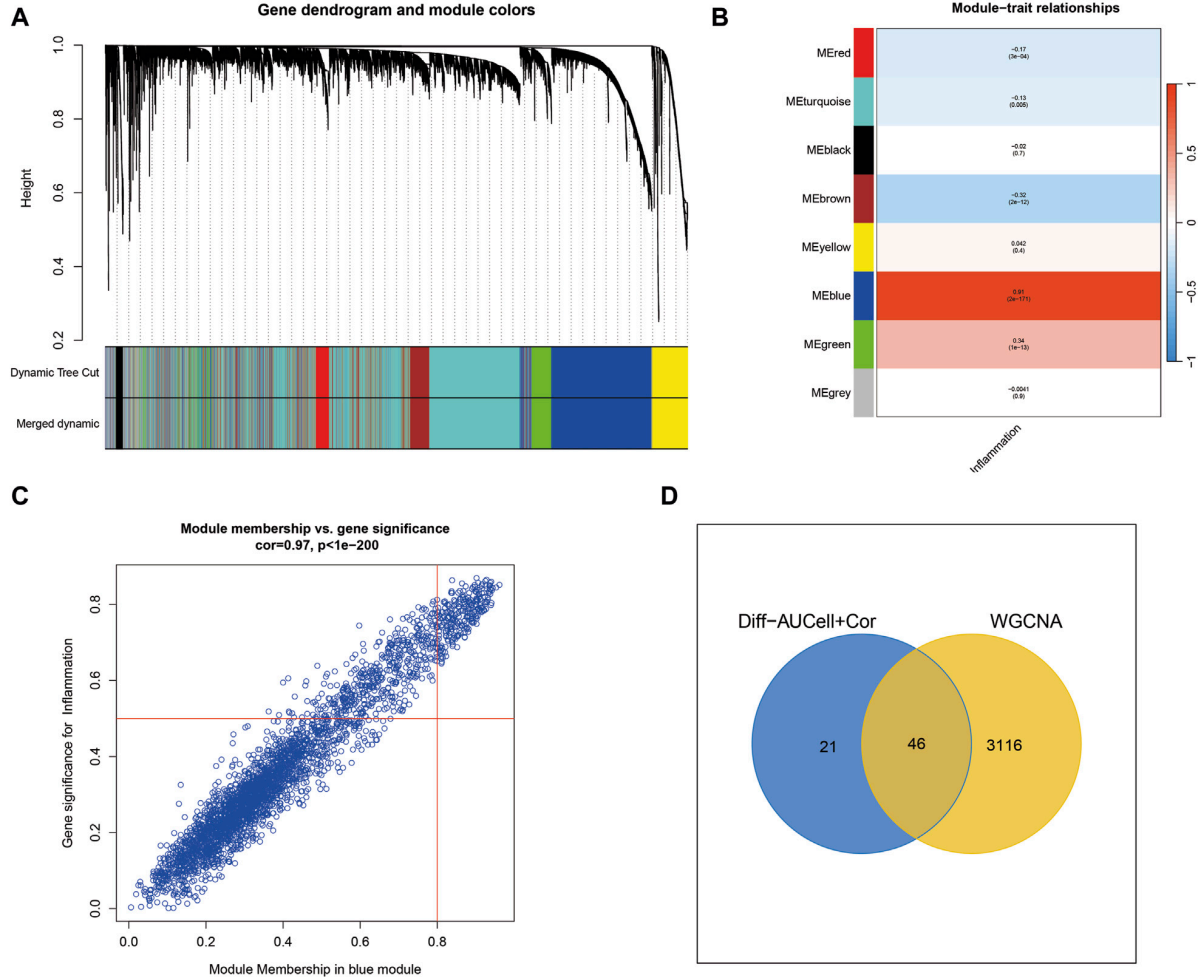
Weighted Gene Co-expression Network Analysis (WGCNA) uncovers key inflammatory modules in cutaneous melanoma. **(A)** WGCNA identifies gene modules, with the blue module showing a strong positive correlation with inflammation-related traits. **(B,C)** The blue module contains 3,162 genes highly associated with inflammation. **(D)** Intersection of genes from WGCNA with those from single-cell sequencing analysis yields 46 genes for machine learning models.

WGCNA analysis of cutaneous melanoma revealed the clustering of various genes into distinct modules, and their relationships with inflammatory phenotypes were assessed. Notably, the blue module exhibited the most robust positive correlation with inflammatory phenotypes, as illustrated in Figures 2A–C. Consequently, a total of 3,162 genes within the blue module were identified by WGCNA as highly associated with inflammation. Subsequently, the 3,162 genes obtained from WGCNA were intersected with the 67 genes acquired through single-cell sequencing analysis, resulting in the identification of 46 genes for the construction of machine learning models (Figure 2D).

### Machine learning is used to construct the optimal prognostic model

**FIGURE 3:**
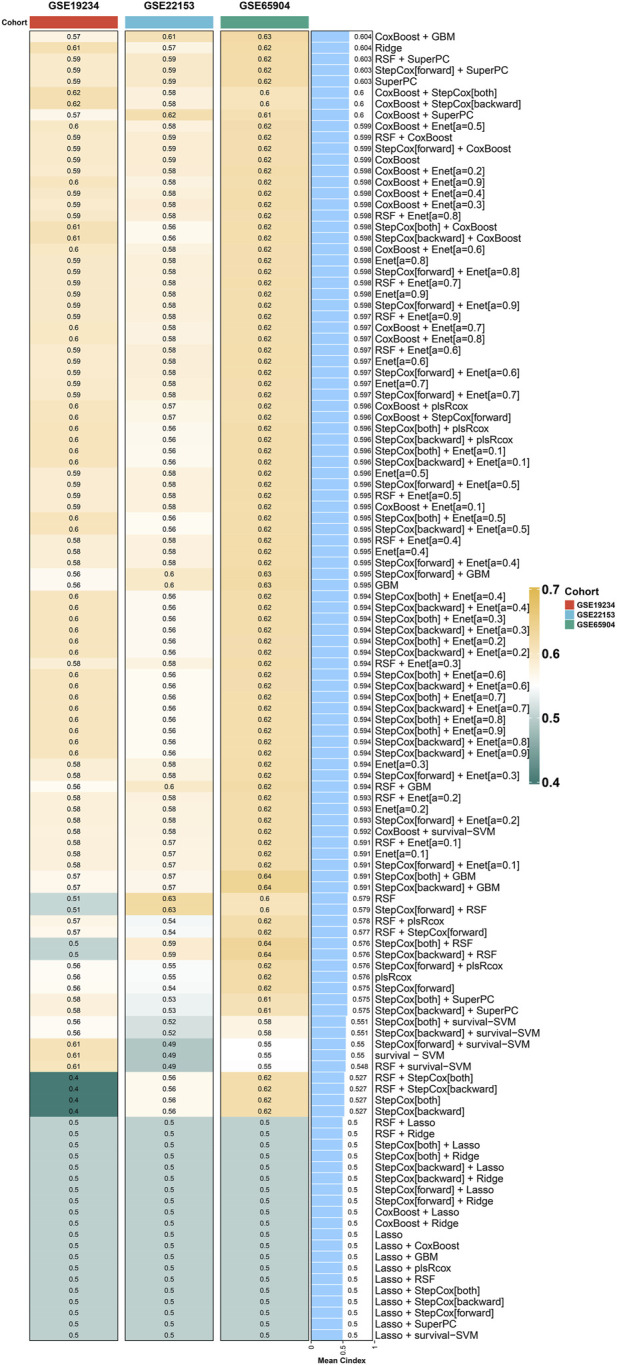
Machine learning models for prognosis in cutaneous melanoma. CoxBoost + GBM method yields the most significant prognostic model with a C index of 0.604.

Following the acquisition of the aforementioned 46 genes, the identification of prognostic-related genes commenced with univariate COX regression analysis. Subsequently, a prognostic model was constructed using a combination of machine learning algorithms, including RSF, COXBoost, Enet, GBM, Lasso, plsRcox, Ridge, StepCox, and survival-SVM. After thorough evaluation, it was determined that the CoxBoost + GBM method yielded the most significant prognostic model, with a C index of 0.604 (Figure 3). This prognostic model consists of six genes, namely, FCGR2A, TLR2, STAT1, IFIT2, LAP3, and PARP14.

### Survival analysis of the prognostic model

**FIGURE 4:**
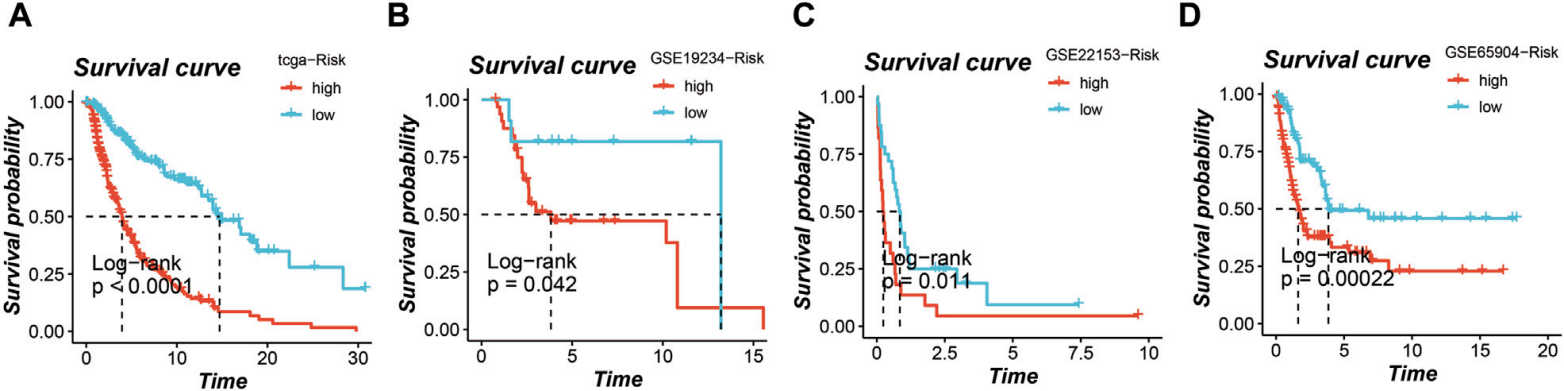
Survival analysis based on the prognostic model in multiple cohorts. High-risk and low-risk groups stratified by the model consistently show poorer prognosis in the **(A)** TCGA cohort, **(B)** GSE19234 cohort, **(C)** GSE22153 cohort, and **(D)** GSE65904 cohort.

In the TCGA cohort, GSE19234 cohort, GSE22153 cohort, and GSE65904 cohort, the application of this prognostic model enabled the stratification of all patients into high-risk and low-risk groups. Notably, across all four cohorts, a distinctly poorer prognosis was consistently observed in the high-risk group, as depicted in Figures 4A–D (*p* < 0.05).

### Nomogram construction and evaluation

**FIGURE 5:**
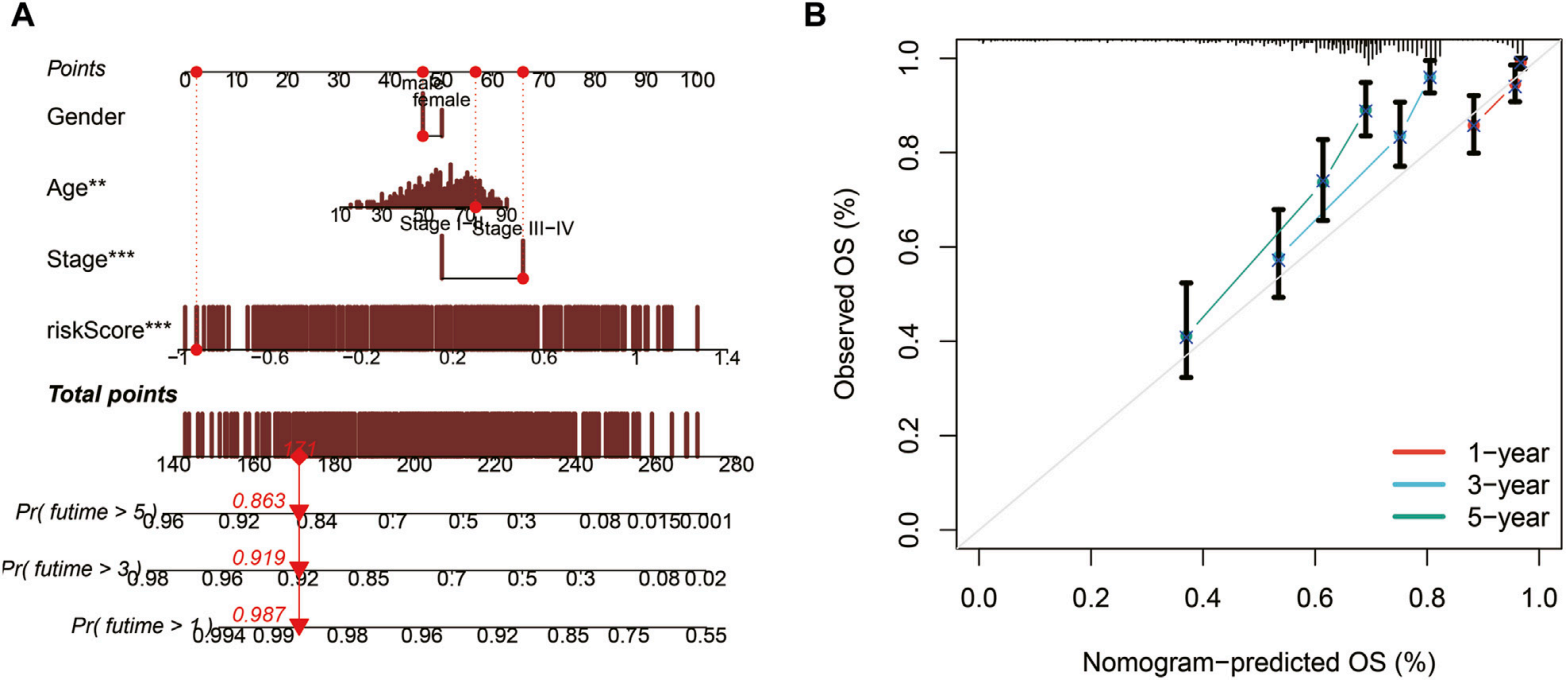
Nomogram construction and evaluation. **(A)** A nomogram integrating risk scores, gender, age, and stage predicts survival probabilities at 1, 3, and 5 years. **(B)** Calibration curves demonstrate the accuracy of the nomogram.

Utilizing TCGA data, a nomogram was developed to assess the 1, 3, and 5-year survival probabilities of patients by incorporating their risk scores, gender, age, and stage. The nomogram demonstrated that the estimated survival rates for patients at 1, 3, and 5 years were 0.987, 0.919, and 0.863, respectively, as illustrated in Figure 5A. The calibration curve further confirmed the accurate performance of the nomogram, as depicted in Figure 5B.

**FIGURE 6:**
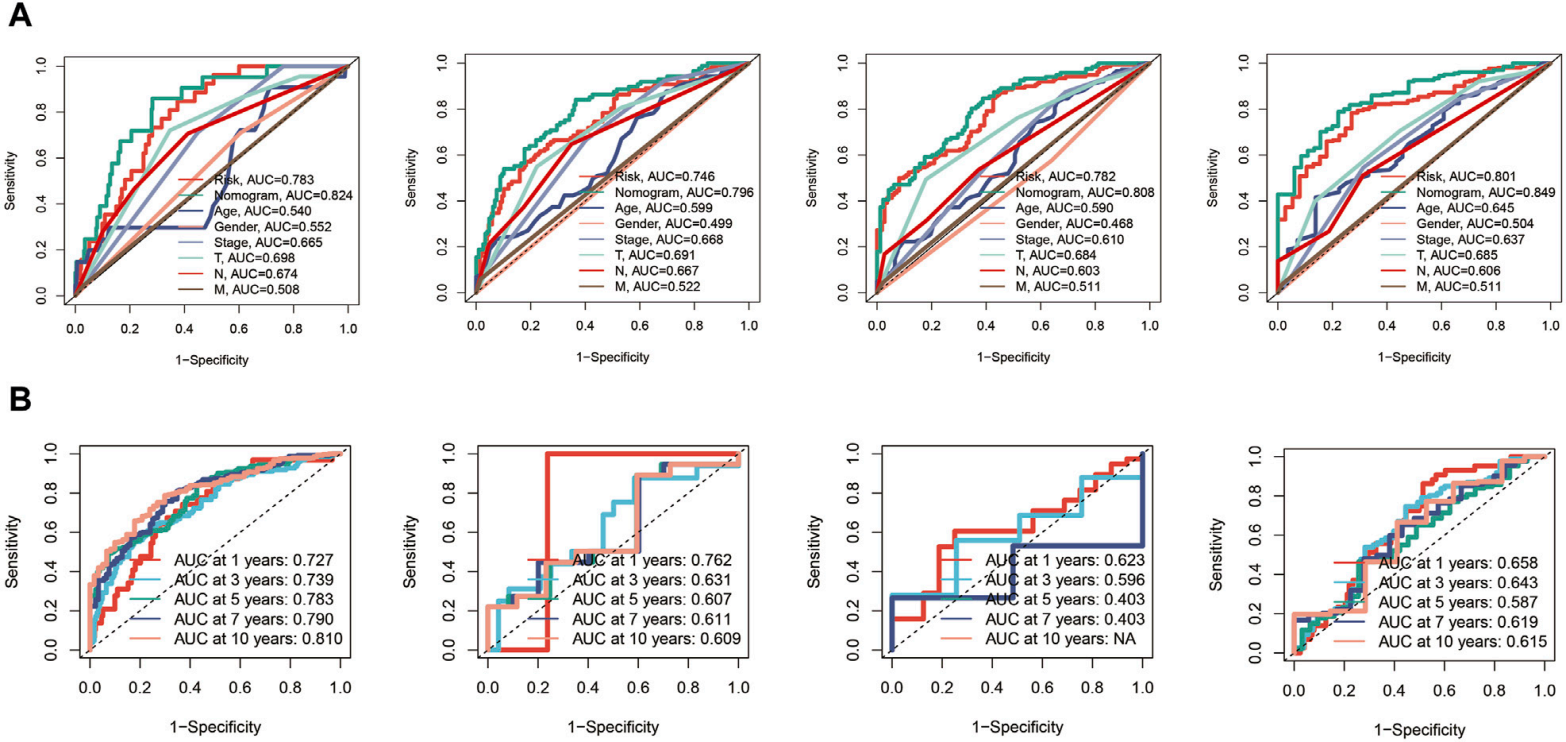
Receiver Operating Characteristic (ROC) curves for the prognostic model in the different cohorts. **(A)** The risk score and nomogram exhibit higher AUC values compared to other clinical indicators for 1, 3, 5, and 7-year survival predictions in TCGA cohort. **(B)** Time-ROC curves in TCGA, GSE19234, GSE22153, GSE65904 cohorts.

Subsequently, ROC curves were generated for 1, 3, 5, and 7-year survival predictions within the TCGA cohort. It was observed that both the risk score and the nomogram exhibited higher AUC values compared to other clinical indicators, affirming the robust predictive capabilities of the prognostic model and the nomogram, as depicted in Figure 6A. Furthermore, time-ROC curves spanning 1, 3, 5, 7, and 10 years were constructed to assess the accuracy of the prognostic model in the TCGA cohort, GSE19234 cohort, GSE22153 cohort, and GSE65904 cohort, demonstrating the model’s effectiveness, as illustrated in Figure 6B.

### Immune cell infiltration, tumor mutation load, and immunotherapy evaluation in the tumor microenvironment

**FIGURE 7:**
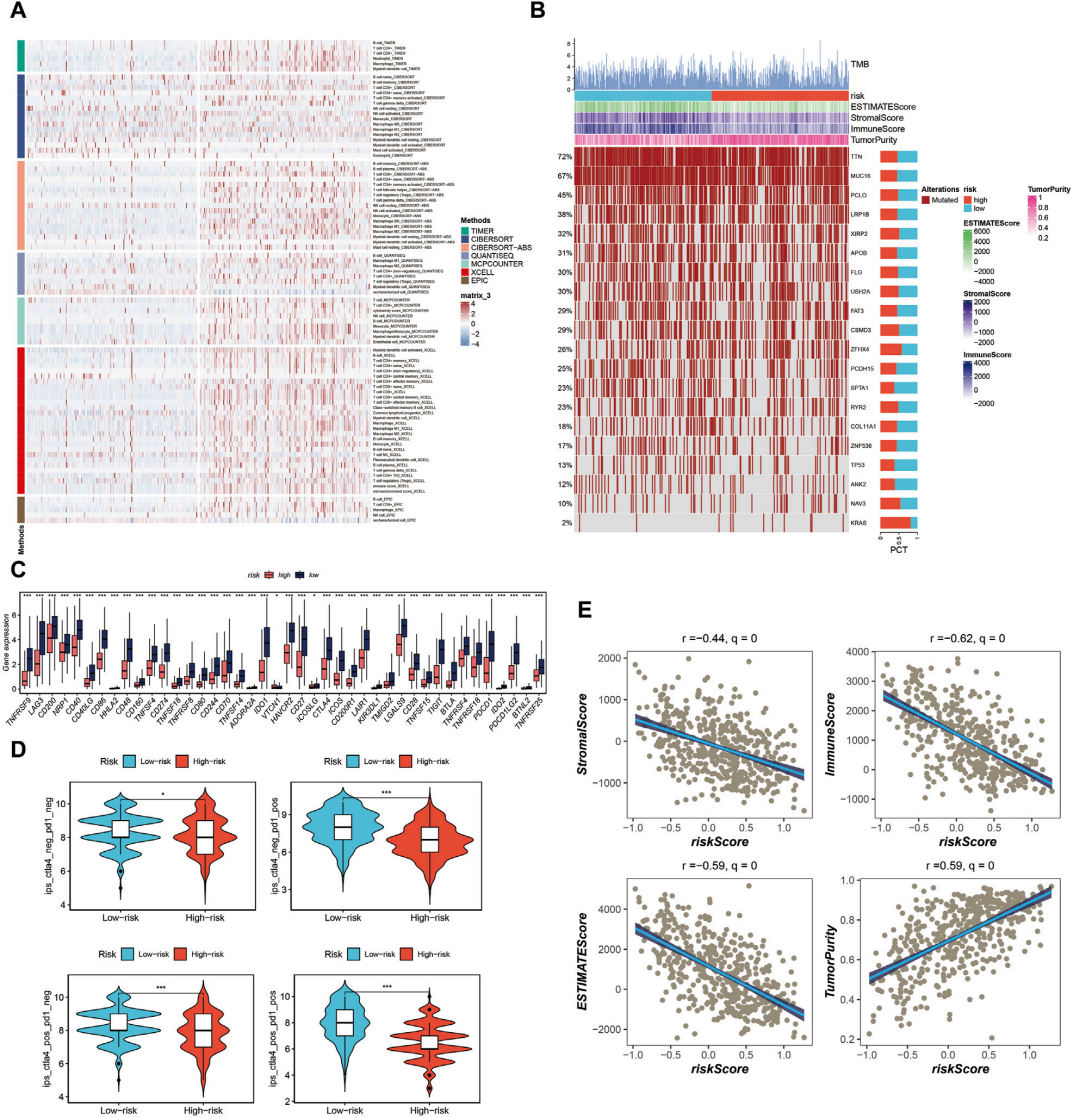
Analysis of immune cell infiltration, tumor mutation load, and immunotherapy response in the tumor microenvironment. **(A)** Immune cell infiltration landscape showing potential higher infiltration in the low-risk group. **(B)** Genes mutation landscape. **(C)** Elevated expression of immune checkpoint-related genes in the low-risk group. **(D)** TCIA scores indicating potential better immunotherapy response in the low-risk group. **(E)** Correlation of risk score with stromal score, immune score, ESTIMATE score, and tumor purity.

In our analysis, we initially constructed the immune cell infiltration landscape, distinguishing between the high-risk group (left) and the low-risk group (right) as shown in Figure 7A. Notably, it became evident that the low-risk group potentially exhibited a higher level of immune cell infiltration. Further exploration of the differences in Tumor Mutational Burden (TMB) between these two groups unveiled genes with the highest mutation frequency, as illustrated in Figure 7B. Investigating immune checkpoint-related genes, we observed that the low-risk group displayed elevated expression levels of immune checkpoint-related genes, suggesting a potential advantage in terms of immunotherapy responsiveness, as indicated in Figure 7C. Additionally, utilizing the TICA score, we found that the low-risk group had a higher TCIA score, potentially indicating a more favorable immunotherapy response, as depicted in Figure 7D. Various scores related to the tumor microenvironment revealed that the risk score exhibited a negative correlation with stromal score, immune score, and ESTIMATE score, while displaying a positive correlation with tumor purity (Figure 7E) (*p < 0.05, ***p < 0.001).

### The key gene STAT1 was found in protein-protein interaction network and verified by cell experiment

**FIGURE 8:**
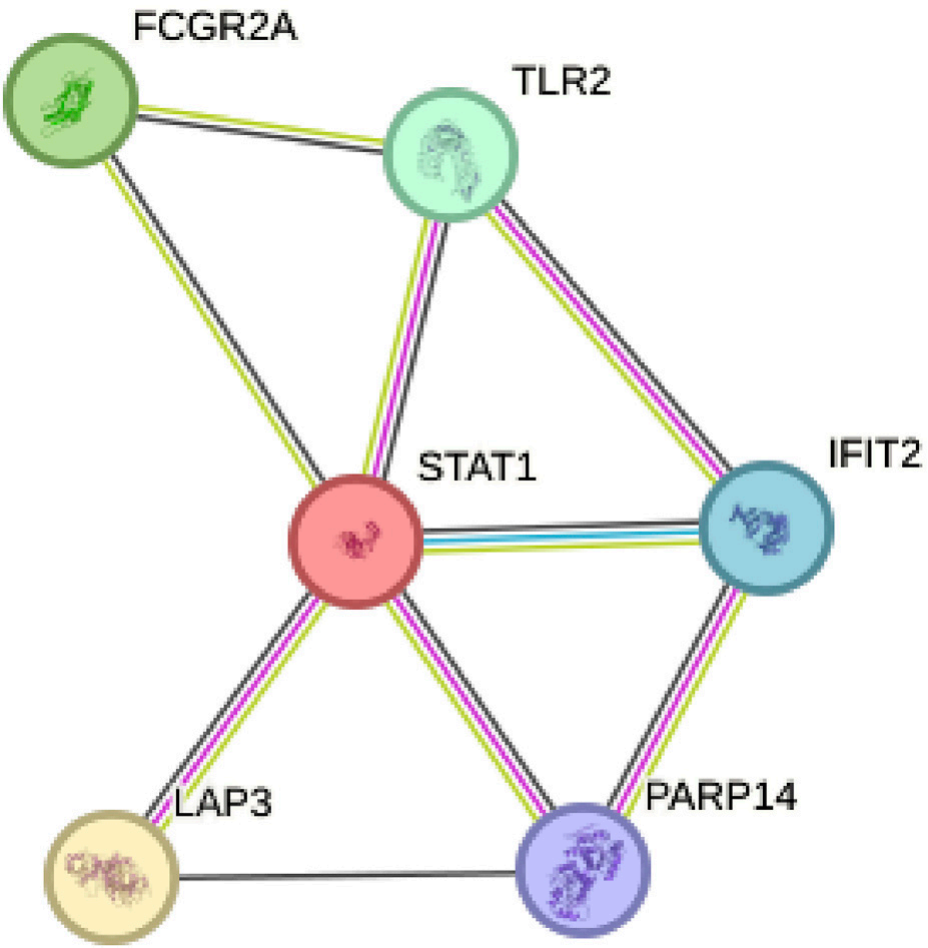
Protein-Protein Interaction (PPI) network identifies STAT1 as a hub gene. STAT1 occupies a central position within the network among the six model genes.

**FIGURE 9:**
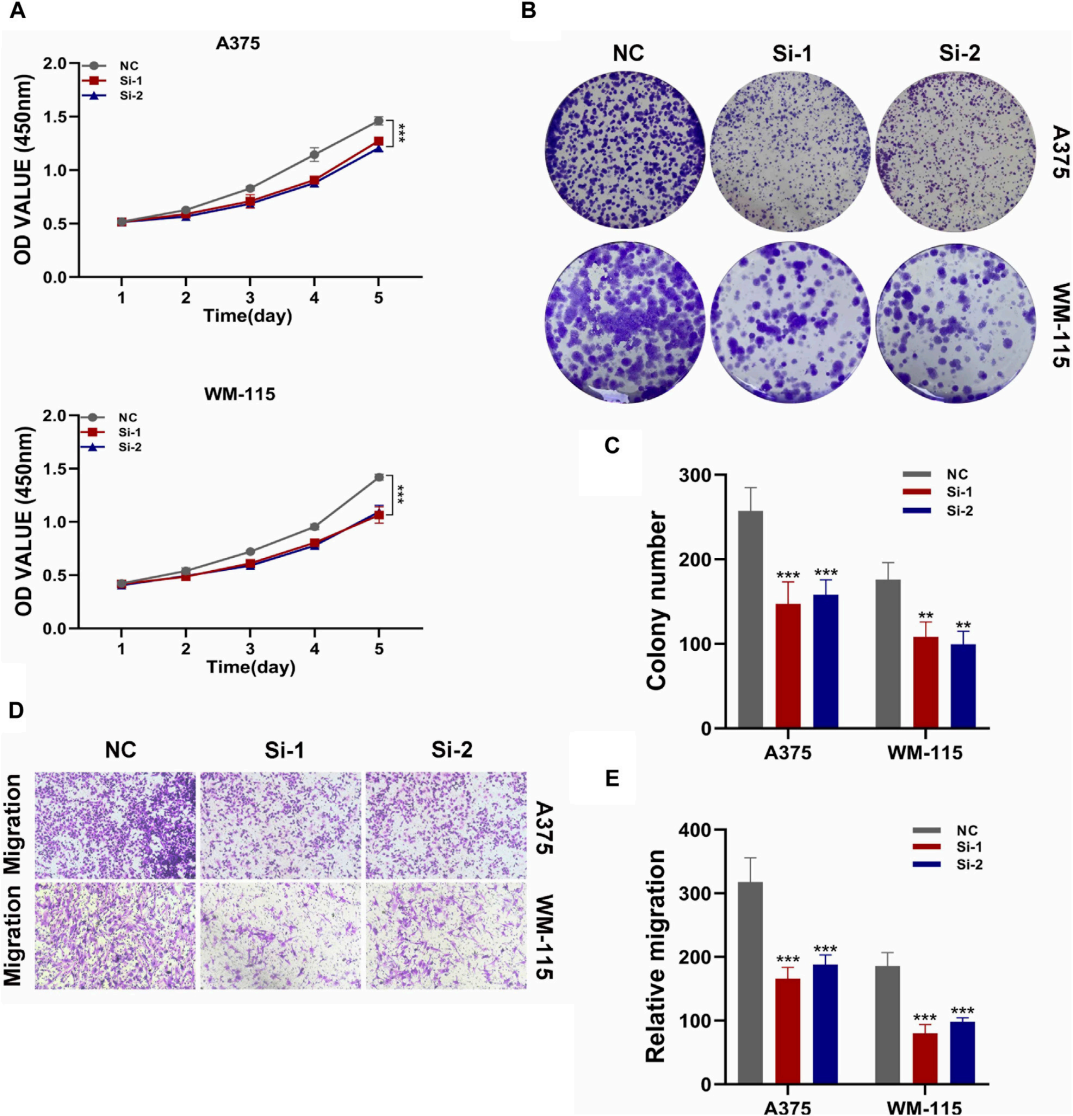
Validation of STAT1 function through cellular experiments. **(A)** CCK-8 experiment shows decreased cell activity after STAT1 knockdown. **(B,C)** Clonal formation assays reveal reduced colony formation ability upon STAT1 knockdown. **(D,E)** Transwell experiments demonstrate decreased cell migration ability following STAT1 knockdown. (*p < 0.05, **p < 0.01, ***p < 0.001).

By constructing a Protein-Protein Interaction (PPI) network, our analysis revealed that among the six model genes, STAT1 occupied a central position within the network, suggesting it may have a crucial hub role, as depicted in Figure 8. Consequently, we chose to validate the function of STAT1 in cutaneous melanoma through cellular experiments. First, the Cell Counting Kit-8 (CCK-8) experiment demonstrated a significant decrease in the activity of A375 and WM-115 cell lines following STAT1 knockdown, as shown in Figure 9A(***p < 0.001). Furthermore, clonal formation assays indicated a marked reduction in colony formation ability for both A375 and WM-115 cell lines after STAT1 knockdown, as illustrated in Figures 9B, C (**p < 0.01, ***p < 0.001). Transwell experiments revealed a significant decrease in the migration ability of A375 and WM-115 cell lines upon STAT1 knockdown, as depicted in Figures 9D, E (***p < 0.001).

## Discussion

In recent years, significant progress has been made in advancing our understanding of the cutaneous melanoma tumor microenvironment (TME) (Bagaev et al., 2021; Hodis et al., 2022; Zhou et al., 2022). This has resulted in increased illumination of the complex interplay between disease progression and inflammation (Denk and Greten, 2022). Melanoma, a formidable malignancy prone to metastasis, has long been linked to chronic inflammation within its microenvironment (Zhang et al., 2020). Progress in research related to inflammation has revealed a crucial aspect of melanoma biology, holding profound implications for both diagnosis and treatment. In this discussion, we will delve into the latest insights concerning the role of inflammation in the melanoma TME and the potential for transformation through the integration of multiomics analysis, machine learning, and functional validation in comprehending and managing this challenging disease.

In this study, a groundbreaking approach was employed, combining single-cell sequencing data with bulk transcriptome data to investigate the heterogeneity and prognostic significance of inflammation in cutaneous melanoma for the first time. The pivotal aspect of single-cell sequencing lies in the identification of various cellular clusters within the tumor microenvironment. This underscores the inherent cellular heterogeneity present in cutaneous melanoma and emphasizes the intricate interplay between inflammation and the progression of the tumor. The elevated inflammation-related scores observed in NK cells, T cells, and macrophages indicate the central role played by these immune cells in shaping the melanoma microenvironment. Comprehending this diversity of cells and the inflammatory context is essential for the development of targeted therapies and immunotherapies.

Weighted Gene Coexpression Network Analysis (WGCNA) reveals gene modules strongly associated with inflammation, providing a systems-level view of molecular interactions driving the inflammatory response in melanoma. Particularly, the blue module stands out as a potential source of key genes contributing to inflammation. These findings deepen our understanding of molecular mechanisms underlying melanoma and offer potential targets for therapeutic intervention aimed at modulating the inflammatory response.

Developing a robust prognostic model using machine learning algorithms represents a significant advancement in clinical management of cutaneous melanoma. By integrating diverse genomic data, this model offers a more precise and personalized approach to prognosis prediction. It has the potential to aid clinicians in stratifying patients into risk groups, enabling tailored treatment strategies. This personalized approach may lead to more effective therapies and improved patient outcomes.

Consistent validation of the prognostic model across multiple cohorts highlights its reliability and generalizability. The nomogram derived from this model is a valuable tool for estimating patient survival probabilities, offering clinicians an accessible means of assessing prognosis. Superior performance compared to traditional clinical indicators underscores its potential for integration into routine clinical practice, ultimately enhancing patient care.

Analyzing immune cell infiltration, Tumor Mutational Burden (TMB), and immune checkpoint-related genes within the tumor microenvironment provides crucial insights into potential immunotherapeutic strategies. Higher immune cell infiltration, increased expression of immune checkpoint-related genes, and a more favorable TCIA score in the low-risk group suggest that these patients may be more responsive to immunotherapy. This finding has significant implications for treatment decisions, guiding the selection of immunotherapeutic approaches for melanoma patients.

Validation of STAT1 as a central hub gene in the protein-protein interaction network, coupled with functional experiments, underscores its potential as a therapeutic target in cutaneous melanoma. The significant impact of STAT1 knockdown on various cellular functions highlights its potential as a target for future therapeutic interventions. Targeting genes like STAT1 could lead to the development of more effective treatments that directly impact melanoma cell behavior.

In conclusion, our study’s findings provide a deeper understanding of cutaneous melanoma’s complexity and its relationship with inflammation. These insights have direct implications for diagnosis and treatment. Prognostic model and nomogram offer a practical means of improving patient care through personalized prognosis assessment. Additionally, insights into the tumor microenvironment and validation of key genes like STAT1 open avenues for development of targeted therapies, immunotherapies, and potential biomarkers to guide clinical decision-making in cutaneous melanoma management. However, there are some limitations to our study. All sequencing data came from public databases, and no patient data and survival data from our center were analyzed. We will make additions in the future.

## Data Availability

The data presented in the study are deposited in the GEO repository, accession number GSE115978, GSE19234, GSE22153, and GSE65904.
