# Supplementary material for: Creating a multifaceted prognostic model for cutaneous melanoma: the convergence of single-cell and bulk sequencing with machine learning
Source: Front Cell Dev Biol. 2024 May 6;12:1401945. doi: 10.3389/fcell.2024.1401945 (PMC11102988; doi:10.3389/fcell.2024.1401945)
Supplement: Supplementary file 1 [file Table2.DOCX]

| **Oligonucleotides** | **Nucleotide sequence (5'-3')** |
| --- | --- |
| **siRNA** |  |
| Scramble control | GCUUCGCGCCGUAGUCUUA |
| Si-STAT1-1 | GAACAGAAATACACCTACGAA |
| Si-STAT1-2 | CCCTGAAGTATCTGTATCCAA |
|  |  |
| **Primer** |  |
| GAPDH | GGCCTCCAAGGAGTAAGACC (forward) |
|  | AGGGGAGATTCAGTGTGGTG (reverse) |
| STAT1 | GTTATGGGACCGCACCTTCA (forward) |
|  | TCATTCACATCTCTCAACTTCACA (reverse) |
|  |  |

**Table S2. Oligonucleotides used in research**
